# Lion lords and sharing hyaenas: Carnivore guild dynamics around elephant carcasses

**DOI:** 10.1002/ece3.11373

**Published:** 2024-05-05

**Authors:** Terry‐Lee Honiball, Robert S. Davis, Liyabona Ntlokwana, Jan A. Venter

**Affiliations:** ^1^ Department of Conservation Management, Natural Resource Science and Management Cluster, Faculty of Science Nelson Mandela University George South Africa

**Keywords:** carnivore coexistence, fatal attraction, interspecific competition, niche partitioning

## Abstract

Megaherbivore carcasses represent sporadic but energetically rewarding resources for carnivores, offering a unique opportunity to study coexistence dynamics between facultative scavengers. South African fenced protected areas, such as Madikwe Game Reserve (Madikwe hereafter), host viable populations of large carnivores and high densities of elephants, *Loxodonta africana*. However, high carnivore densities can lead to potentially fatal interspecific encounters and increased competition, particularly around high‐quality trophic resources. This study explores the temporal partitioning and co‐detection strategies of carnivores at six elephant carcasses in Madikwe, aiming to understand how the increased carrion biomass available at elephant carcasses influences coexistence dynamics. Camera traps were deployed to monitor carcasses during two periods (2019 and 2020), revealing occurrences of six carnivore species. Carnivores, particularly black‐backed jackals, *Lupulella mesomelas*, (hereafter jackal), lions, *Panthera leo*, and spotted hyaenas, *Crocuta crocuta*, comprised 56.7% of carcass observations, highlighting their pivotal roles in scavenging dynamics. Pairwise co‐detection analysis demonstrated consistent association and shared peak activity periods between jackals and spotted hyaenas, indicating potential resource sharing. However, the minimal co‐detection rates between lions and other carnivores highlight their resource domination. There was some evidence of temporal partitioning between carnivores, with most species exhibiting earlier peaks in nocturnal activity to avoid temporal overlap with lions. This study emphasises the importance of elephant carcasses in the diet of multiple species and coexistence techniques utilised to exploit this ephemeral resource. As fenced protected areas become crucial for conserving intact carnivore guilds globally, further research into carnivore behavioural adaptations at carcasses is recommended to shed light on their coexistence strategies.

## Graphical abstract

Megaherbivore carcasses represent sporadic but energetically rewarding resources for carnivores and it is poorly understood how these carrion resources impact interspecific interactions. We studied elephant carcasses in Madikwe Game Reserve, South Africa, to investigate temporal partitioning and co‐occurrence between the carnivore guild. Co‐occurrence was high between some carnivores, suggesting aggressive interactions may be reduced by abundant food resources.

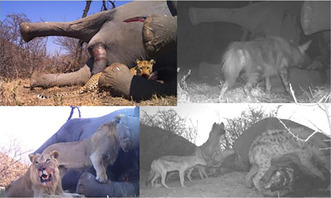

## 1 INTRODUCTION

The functional roles of carnivores are diverse, allowing for coexistence in the same environment (Davis et al., 2018). To facilitate coexistence, species have evolved strategies to compete for shared resources (Davis et al., 2018). Such strategies include spatial and temporal partitioning or adaptions in specific resource use (Monterroso et al., 2020). Spatially, subordinate carnivores may adjust their territories to less favourable habitats to avoid competition (Elbroch & Kusler, 2018). Temporally, carnivores may adjust their circadian activity to reduce the likelihood of encountering competitors (Zhao et al., 2020). Additionally, carnivores may broaden or narrow their dietary niche to reduce competitive pressures (Jonathan Davies et al., 2007). In different ecosystems, the effect of apex predators, defined here as the dominant predator in a system, on subordinate carnivores has been extensively studied, providing insight into coexistence strategies (Davis et al., 2018; Elbroch et al., 2020; Sivy et al., 2018). Most commonly, apex predators exert top‐down pressure on subordinate carnivores resulting in shifts in how these species interact with their environment (Elbroch & Kusler, 2018; Hilborn et al., 2018). Even where two apex predators co‐occur, typically one will shift into a less dominant role to reduce conflict. In Scandinavia and North America, when wolves, *Canis lupus*, experience top‐down pressure from brown bears, *Ursus arctos*, they reduce their kill rates, which has direct energetic impacts on these predators (Tallian et al., 2022). Furthermore, through top‐down pressure tigers, *Panthera tigris*, displace leopards, *Panthera pardus*, forcing leopards into suboptimal habitats (Odden et al., 2010).

Top‐down pressure on subordinate carnivores is amplified when space is limited, as species then need to adopt coexistence techniques alternative to spatial avoidance (Hanby & Bygott, 1995). As urbanisation increases, areas considered optimal habitat decrease. Fenced protected areas are an extreme example of the reduction of optimal areas forcing species to co‐occur, thus allowing the opportunity to understand the coexistence of competing species when space is limited by physical barriers (Parker et al., 2021; Thapa et al., 2021). As tourism is a primary revenue source for fenced protected areas, these areas often stock a high diversity of charismatic species at elevated densities to satisfy tourist desires (Rylance et al., 2017). Tourism, through photographic safaris, thrives when sightings of species, such as elephants, *Loxodonta africana*, white rhinoceros, *Ceratotherium simum*, and lions, *Panthera leo*, are frequent (Mangachena & Pickering, 2023). Thus, providing incentive to allow such charismatic species densities to remain high (Trouwborst et al., 2016). This approach to fenced protected area management results in multiple apex predators in the same area, forcing adaptions in dominance and species shifting to more subordinate roles (Prugh & Sivy, 2020). Spotted hyaenas, *Crocuta crocuta*, and lions are two dominant carnivores that occur in many fenced protected areas and coexistence strategies between these two species are often a complex balance between facilitation and competition (Périquet et al., 2015). Interactions between lions and spotted hyaenas are often negative and potentially fatal (Périquet et al., 2015; Trinkel & Kastberger, 2005). Kleptoparasitism of kills are frequent between the two species, making hunting a costly source of food compared to scavenging (Périquet et al., 2015). Thus, to mitigate some of the energetic costs of hunting, carnivores often supplement their diets with carrion (Amorós et al., 2020).

Due to legislation (National Environmental Management: Biodiversity Act, 2004) and limited available habitat affecting management capabilities in fenced protected areas, charismatic megaherbivores (>500 kg) such as elephants can reach densities beyond typical carrying capacities (Selier et al., 2016). Addo Elephant National Park, South Africa, had an estimated carrying capacity of 0.1–0.5 elephants per km^2^ in 2006 but maintained a stocking density up to 4 elephants per km^2^ (Gough & Kerley, 2006). A similar scenario prevails in Madikwe Game Reserve (Madikwe hereafter), South Africa, which maintains a stocking density within the higher ranges of elephant stocking densities for fenced reserves at 1.9 elephants per km^2^ in 2018 (Szott et al., 2019). Where megaherbivores occur at high densities, they become a potentially more frequent source of carrion when individuals die due to age, health or territorial conflicts, thus providing an ephemeral food source (Loveridge et al., 2006). When considering the elevated predator pressure on resources in fenced protected areas, carrion provided by elephant carcasses could provide relief from the energetic costs associated with coexistence of carnivores.

Although providing relief, elephant carcasses may also attract many scavenging species, creating a point of increased interactions, particularly between dominant and subordinate predators (Morris et al., 2023). Thus, understanding the role of megaherbivore carcasses in the diet of apex predators such as lion and spotted hyaena is important to provide insight into coexistence strategies adopted at shared resources. Furthermore, the consumption of carrion is important for mesocarnivores in many environments (Prugh & Sivy, 2020), and there is a need to understand how mesocarnivores utilise these ephemeral resources in the presence of dominant carnivores (Sievert et al., 2023).

Therefore, the aim of this study was to (1) gain insight into how spotted hyaenas and lions utilise ephemeral food resource such as elephant carcasses, and (2) to assess what impact dominant carnivores, such as lions and spotted hyaenas, have on temporal activity of mesocarnivores at elephant carcasses. We then compare our findings to existing literature from Madikwe, occurring at the same time, to understand the role of elephant carcasses in shifts of behavioural responses by carnivores. We predicted lions would display dominance around elephant carcasses. Furthermore, we predicted that there would be temporal partitioning of carcass use between lions and spotted hyaenas as they are direct competitors (Périquet et al., 2015). Where spotted hyaenas are subordinate to lion, in terms of the trophic hierarchy, such as is the case in Madikwe, we predict the relaxation of temporal partitioning of resource use of megaherbivore carcasses between spotted hyaenas and other mesopredators. Finally, we predicted co‐detection of species to increase as the carcasses aged as carrion became more accessible to a wider variety of species.

## 2 MATERIALS AND METHODS

### 2.1 Study site

Madikwe is a 650 km^2^ protected area situated in the North‐West Province of South Africa and is largely comprised of arid savanna enclosed by electrified predator‐proof fencing (Mucina & Rutherford, 2006). Madikwe was established in 1991, and in 1994, among other herbivores, elephants were introduced. Since then, the population has grown substantially to 1.9 elephants per km^2^ in 2018 (Davies, 2000; Szott et al., 2019). The reintroduction of lions, cheetahs, *Acinonyx jubatus*, African wild dogs, *Lycaon pictus*, and spotted hyaenas took place in 1997 (Davies, 2000). Brown hyaenas, leopards and various mesocarnivores naturally occurred in the park and populations of these species continue to persist (Davies, 2000). At the time of this study, Madikwe supported an intact African large carnivore guild, where exact populations were known through intensive management of lions (33 individuals), cheetahs (six individuals) and African wild dogs (12 individuals). Honiball (2021) estimated populations at the time for spotted hyaenas (81 ± 0.78 individuals), brown hyaenas (92 ± 1.39 individuals) and leopards (24 ± 0.23 individuals).

### 2.2 Field sampling

In 2019, two different elephant mortalities resulted in the availability of carcasses (one in August, at the end of the dry season and one in November, at the beginning of the wet season). In 2020, four carcasses became available between August and September (end of dry season, early Spring). Carcasses were located opportunistically during ranger patrols. Carcasses deemed suitable for monitoring were <48 h old. Carcass age was estimated by a lack of vulture activity, absence of maggots or other signs of the onset of substantial decomposition. Suitable elephant carcasses were monitored with a black flash camera trap (Cuddeback C3 with a 16 GB memory card) and were monitored until only skin and bones remained (16 ± 4 days). A single camera trap was deployed at each carcass, consequently some potential detections may have been missed. Cameras were fixed to metal poles to allow for selective placement. Cameras were placed at a height of 60 cm above the ground. The pole was positioned at least 4 m from the carcass facing the softer tissue of the carcass as this is where scavengers focus their efforts when consuming a carcass (White & Diedrich, 2012).

Cameras were programmed to take four images per trigger with a 1‐min delay between trigger events. Four images allowed for extended insight into activity at the time of the trigger. A one‐minute delay may result in individuals being missed as they move past the camera, however, this time delay was deemed sufficient to provide insight into the activity of species around the carcasses. Each image sequence was considered a single observation, considering all individuals across the image sequence to be part of the same observation. The date and time associated with each image sequence was extracted from the first image of the sequence. Species were manually identified and listed in Microsoft Excel before exporting to R version 4.1.1 for analysis (R Core Team, 2021). As avian scavengers were not the focus of this study, we grouped all avian species into the category “bird”.

### 2.3 Analysis

We determined general circadian activity patterns by estimating kernel density across all six carcasses. Activity peaks were determined by using the time of the highest kernel density estimate for each species across the six carcasses. Circadian activity overlap coefficients were determined using the ‘overlap’ package in R (Meredith & Ridout, 2021). The coefficient of overlap ($\hat{\Delta }$) was estimated on a scale of 0 and 1, where 1 represents the total overlap and 0 represents no overlap. Following Meredith and Ridout (2021), ${\hat{\Delta }}_{4}$ estimators were used for all species apart from leopards, as all species had >50 records. Leopard however had <50 records, therefore ${\hat{\Delta }}_{1}$ was applied for species–leopard combinations. Estimates of “low”, “moderate” and “high” overlap were adapted from Monterroso et al. (2014), whereby low was <50% $\hat{\Delta }$, moderate was 51% < $\hat{\Delta }$ < 75% and high constituted $\hat{\Delta }$ >76%.

We considered co‐detection to be existent when two species were recorded together in the same image sequence. If three or more species were recorded in the same image sequence, co‐detection was determined on a pairwise basis of single species/single species. Co‐detection rates were calculated for each species by determining the number of times a species was recorded in the same image sequence with another species as a function of the total records of the said species. Thereafter, pairwise co‐detection between species pairs was determined to assess which species were recorded together.

As in Sievert et al. (2023), we defined a sampling occasion to be a 24‐h period from midday to midday due to the largely nocturnal behaviour of the most commonly occurring species that utilised carrion. Co‐detection was then determined per sampling occasion. Co‐detection as a function of sampling occasion was visualised using ‘*ggplot*’ (Kassambara, 2013). We then used generalised linear mixed models (GLMMs) with a Poisson distribution to assess how co‐detection count changed as the carcasses aged (sampling occasion increased), while considering carcass as a random effect to account for the differing effects of carcass location or time of carcass occurrence (‘*lme4*’ *package*: Bates et al., 2009). Only lion, spotted hyaena, brown hyaena and jackal were considered in the GLMMs as they each had >50 counts of co‐detection. To determine co‐detection count, the number of times a species was observed with any other species was calculated for each occasion (24 h) to produce a co‐detection count per sampling occasion for each species, at each carcass.

To determine if the length of time a carcass was monitored affected the probability of recording species co‐detection, we used linear models. First, the probability of a species being co‐detected with another species was calculated by determining the number of co‐detections per species over the total number of detections per species for each carcass per sampling occasion. A co‐detection probability was determined in general across all species and then individually for brown hyaena, spotted hyaena, jackal and lion. Linear models were then used to assess if the probability of recording a co‐detection was affected by the length of time a carcass was monitored using the ‘*lm*’ function. Linear models were run for general and species‐specific co‐detection probabilities.

To determine if the presence of a lion or spotted hyaena at a carcass affected the presence of jackals, we used GLMMs with a Poisson distribution. A binary capture history of daily detection for each species was created to determine the frequency at which the focal species utilised a carcass. Jackal detection frequency (response variable) was then modelled as a function of lion detection or spotted hyaena detection while accounting for carcass ID as a random effect.

## 3 RESULTS

### 3.1 Observations and co‐detection

**FIGURE 1:**
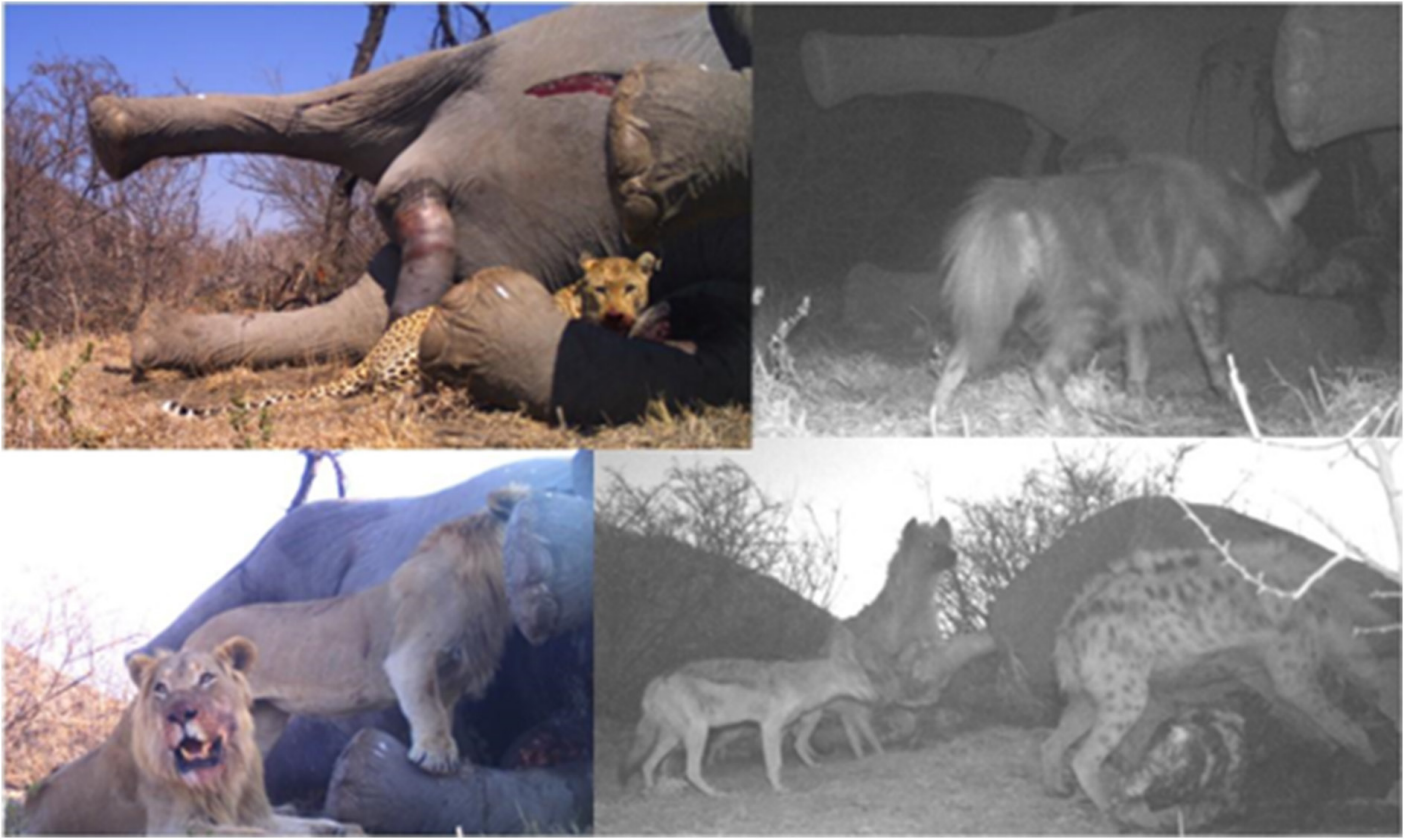
A sample of images of different carnivore species feeding on a single elephant carcass at different stages of decomposition in Madikwe Game Reserve, South Africa. A male leopard, *Panthera pardus*, is pictured top‐left, a brown hyaena, *Parahyaena brunnea*, in the top‐right, two male lions, *Panthera leo*, in the bottom‐left and spotted hyaenas, *Crocuta crocuta*, with black‐backed jackal, *Lupulella mesomelas*, in the bottom‐right.

**FIGURE 2:**
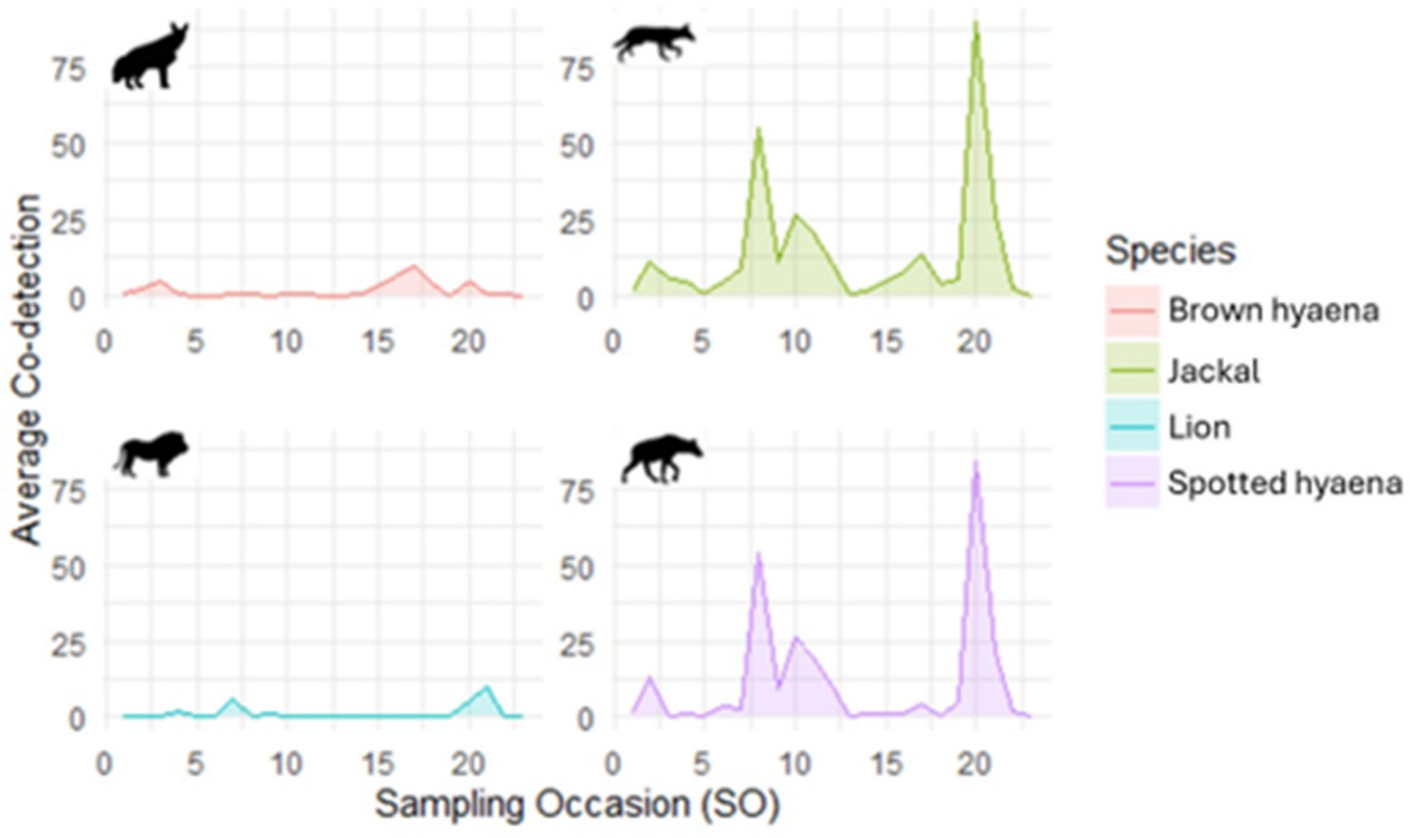
Co‐detection fluctuations are shown for brown hyaena, *Parahyaena brunnea*, jackal, *Lupulella mesomelas*, spotted hyaena, *Crocuta crocuta*, and lion, *Panthera leo*, averaged across six elephant, *Loxodonta Africana*, carcasses over 24 days/sampling occasions. Sampling occasion is defined as a 24‐h period from midday to midday.

Fourteen species were recorded across the six carcasses (Table 1). Carnivora were recorded most frequently at the carcasses and appeared to be the primary consumers of available carrion (Figure 1). Of the species recorded at the carcasses, jackals, spotted hyaenas and lions were the most frequently recorded (*n* = 3708, 3587 and 3330 detections, respectively), while civets, *Civettictus civetta*, were the least frequently recorded (*n* = 2 detections). Jackals and spotted hyaenas had the highest general co‐detection rates (Table 1). The highest pairwise co‐detection rates were recorded between jackals and spotted hyaenas, followed by jackals and brown hyaena, and jackals and lions (Figure 2).

**TABLE 1:** Total observations and co‐detection rates from camera trap data of species across six elephant carcasses in Madikwe Game Reserve (2019–2020).

			Carcass attendance	Total records	Co‐detection rate
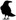	Avian	*Corvus albus*	6	2143	0.07
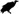		*Gyps africanus*			
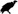		*Torgos tracheliotos*			
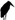		*Leptoptilos crumenifer*			
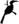		*Tockus leucomelas*			
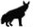	Carnivore	*Parahyaena brunnea*	6	1243	0.05
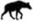		*Crocuta crocuta*	6	3587	0.24
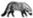		*Civettictis civetta*	1	2	0
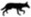		*Lupulella mesomelas*	6	3708	0.28
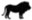		*Panthera leo*	4	3330	0.03
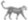		*Panthera pardus*	1	46	0
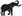	Herbivore	*Loxodonta africana*	5	279	0.001
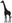		*Giraffa giraffa giraffa*	1	28	0
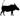		*Phacochoerus africanus*	1	22	0

*Note*: Co‐occurrence was defined as when two species were recorded together in the same trigger event. For co‐detection, a value of ‘1’ indicates that a species was recorded with any other species anytime it was recorded at a carcass, while a value of ‘0’ indicates the species was never recorded with another species. Species silhouettes are not to scale.

Species, such as giraffe, *Giraffa giraffa giraffa*, and civet, were briefly recorded utilising a carcass when no other species were present. The giraffe chewed on the elephant's tusk and a femur. A civet approached the carcass and then left, being recorded on a single occasion.

Species co‐detection increased after the carcass had been in situ for ≥2 weeks (Figure 2: which represents averages of all carcasses while GLMMs considered the effects of individual carcasses). Brown hyaena (*β*_occasion_ = 0.091 ± 0.013; *p* = <.001), lion (*β*_occasion_ = 0.262 ± 0.015, *p* = <.001) and jackal (*β*_occasion_ = 0.08 ± 0.005, *p* = <.001) co‐detection increased significantly as the carcass aged. However, no marked changes in co‐detection were recorded for spotted hyaenas (*β*_occasion_ = −0.001 ± 0.008, *p* = .9).

The variation in the length of time each carcass was monitored (16 ± 4 days) did not affect the probability of observing species co‐detection in general or for individual species (spotted hyaena, brown hyaena, jackal and lion: Appendix 1). Jackal carcass use was not affected by lion's presence (*β*_occasion_ = 0.103 ± 0.182, *p* = .569), however, jackal carcass use increased significantly when a carcass was utilised by spotted hyaenas (*β*_occasion_ = 2.11 ± 0.108, *p* = <.001). Carcass use by lions or spotted hyaenas was insignificantly negatively affected by each other.

### 3.2 Circadian activity patterns

**FIGURE 3:**
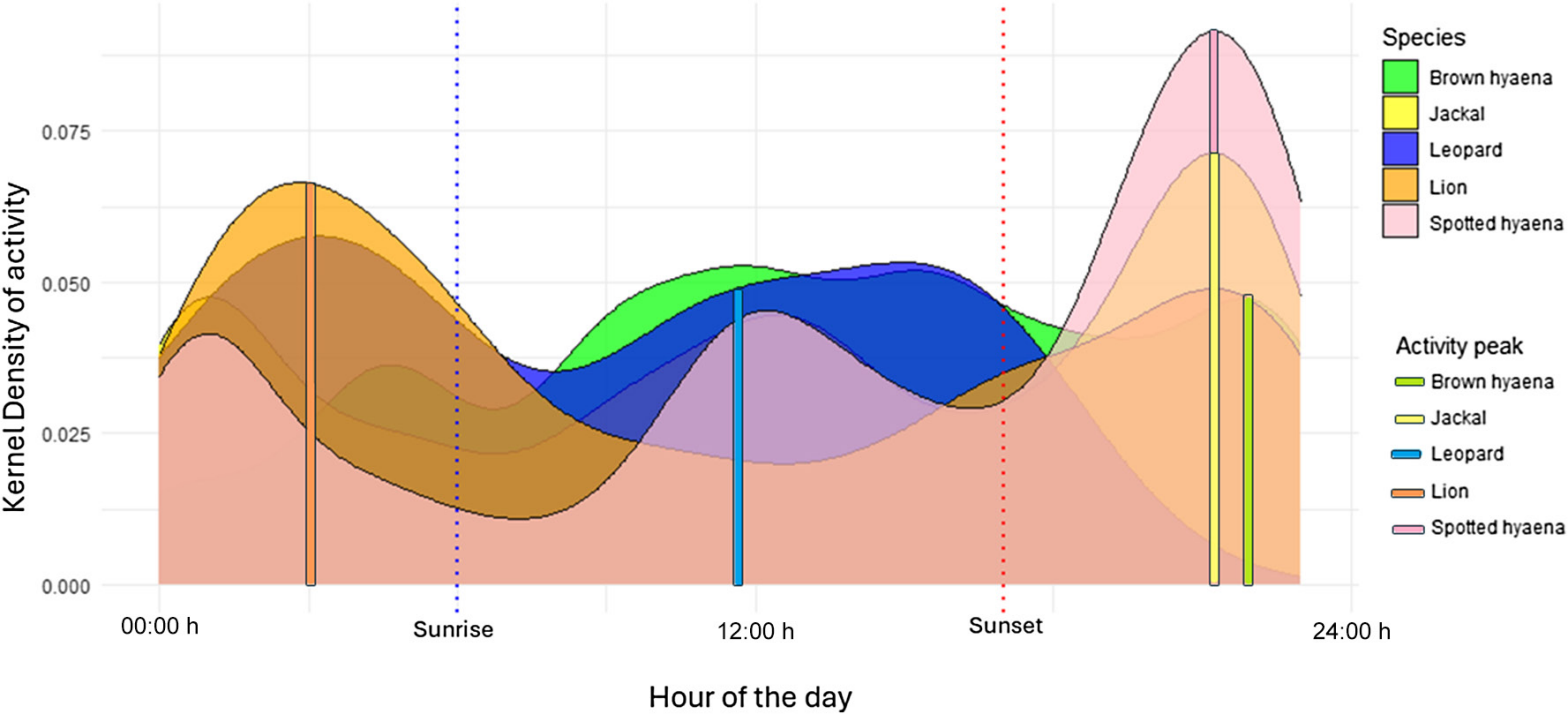
Circadian activity and peak activity periods of selected carnivores measured across six elephant carcasses in Madikwe Game Reserve (2019–2020). Sunrise (06:00 h) and sunset (18:00 h) are represented by vertical dotted lines. Species activity peaks are represented by solid vertical lines.

Circadian activity of carnivores at elephant carcasses in Madikwe showed levels of temporal overlap across all species, however differing activity peaks suggest temporal avoidance (Figure 3). Activity peaks of spotted hyaena, brown hyaena and jackal images occurred between 21:00 h and 22:00 h (Figure 3), while lion activity peaked at 03:00 h, and leopard activity peaked at 11:00 h.

### 3.3 Circadian overlap

Jackals exhibited the highest overlap values with other species. The highest overall circadian overlap was recorded between jackal and spotted hyaena, followed by jackal and lion (Table 2). The lowest circadian overlap was recorded between spotted hyaena and leopard (Table 2).

**TABLE 2:** Circadian activity overlap of carnivores in Madikwe Game Reserve, South Africa, across six elephant carcasses monitored using camera traps in 2019 and 2020. With a value of ‘1’ indicating complete overlap and ‘0’ indicating no overlap.

	Lion	Leopard	Jackal	Brown hyaena	Spotted hyaena
Lion		0.65	0.78	0.73	0.69
Leopard	0.65		0.60	0.67	0.55
Jackal	0.78	0.60		0.77	0.87
Brown hyaena	0.73	0.67	0.77		0.70
Spotted hyaena	0.69	0.55	0.87	0.70	

## 4 DISCUSSION

In this study, we assessed co‐detection and temporal activity of carnivores at six elephant carcasses in Madikwe during 2019 and 2020. We found that although there is circadian activity overlap among all species, co‐detection may be a better predictor of interspecies tolerance and avoidance. Lions entirely dominated carcasses and were rarely recorded with other species, while jackals were frequently recorded with most sympatric carnivores and shared a peak activity period with spotted hyaenas. Species that did not frequently visit carcass sites included leopard, civet, and warthog, *Phacochoerus africanus*. Images of spotted hyaena, jackal and lion constituted 56.7% of the total images recorded at carcasses, suggesting they are responsible for a large proportion of carrion reduction at elephant carcasses. Furthermore, we recorded an interesting incident of giraffe osteophagia directly on an elephant carcass.

Jackals frequently consumed carrion from elephant carcasses in Madikwe and had the highest rates of co‐detection of any carnivore. Notably, jackals shared an activity peak with spotted hyaena and these two species accounted for the majority of co‐detection records. Previous research has found that jackals avoid spotted hyaenas (Comley et al., 2020) but interactions between the two species at carcasses have not been previously studied. Our findings suggest spotted hyaena may tolerate jackals at megaherbivore carcasses, where resources are abundant. Spotted hyaena and jackal have vastly different body sizes, which could limit the perceived level of threat between species and result in increased tolerance (Vissia & Van Langevelde, 2022). Large carcasses may not be a critical food source for spotted hyaenas or jackals, as they can readily access alternative food sources (Humphries et al., 2016; Pereira et al., 2014). The tolerant behaviour recorded for jackals and by jackals in Madikwe emphasises the findings of Welch et al. (2022) which show the site‐specific, adaptive behaviour of jackals.

Our results contrast with those of Morris et al. (2023), who only recorded jackals feeding on one of the two elephant carcasses in Tsavo East National Park, Kenya, and the species was infrequently (*n* = 60 detections) recorded, relative to our observations in Madikwe (*n* = 3708 ~ 6 carcasses). Spotted hyaena can reach higher densities in East African savanna ecosystems (e.g., >100 individuals per 100 km^2^; Höner et al., 2005) and increased clan size may lead to greater rates of resource dominance and reduced tolerance of sympatric carnivores. Sievert et al. (2023) found that spotted hyaena dominated carcass access in Liwonde National Park, Malawi, and were never recorded sharing carcass access with mesocarnivore species. Despite this, in Madikwe, the presence of spotted hyaenas increased the probability of jackal detection twofold, showing that when a spotted hyaena is not the apex predator, they may shift into a more facilitative role. A benefit of dominance is access to prime resources, when spotted hyaenas fall into the role of subordinate, the need to defend lower‐quality resources may not justify the risk of injury. Thus, when spotted hyaenas are subordinate they fulfil a facultative role by opening carcasses up and not defending the resource as lions may in the dominant role.

During the same period in Madikwe, Evers et al. (2022) found, while using a combination of baited (50 kg of carrion) and non‐baited camera traps, that lions and leopards exhibited a moderate to high degree of circadian overlap. Evers et al. (2022) found leopards and spotted hyaena exhibited the highest level of circadian overlap, while our study found the opposite, with spotted hyaena and leopard having the lowest temporal overlap. This may indicate that the likelihood of leopard scavenging behaviour is influenced by carcass size and its associated perceived threat. Both lions and spotted hyaenas are known to increase foraging time at larger carcasses (Amorós et al., 2020; Sievert et al., 2023) and, owing to group size and their larger body mass, both species can exert competitive dominance over leopards for resource access. In this study, male leopards increased their diurnal activity around elephant carcasses, while previous studies have found that leopards, particularly females, increase levels of diurnal activity to avoid competing carnivores (Davis et al., 2021; Havmøller et al., 2020). No female leopards were observed at the elephant carcasses in Madikwe, which emphasises that female leopards may be more risk averse than males. Panda et al. (2023) found similar results for solitary carnivores in India, with striped hyaena (*Hyaena hyaena*) having reduced levels of carrion acquisition in the presence of group‐feeding carnivores such as golden jackals, *Canis aureus*.

**FIGURE 4:**
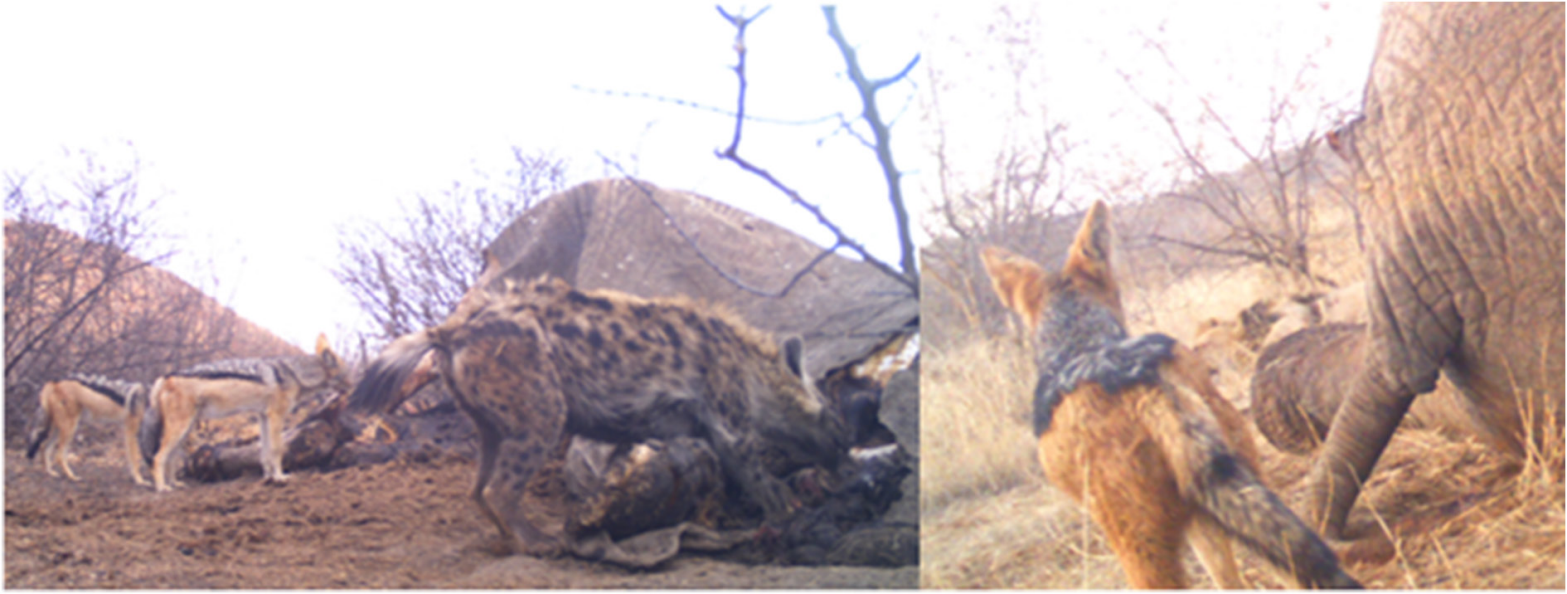
An example of camera trap images at elephant carcasses in Madikwe. The image to the left shows black‐backed jackals, *Lupulella mesomelas*, feeding simultaneously with a spotted hyaena, *Crocuta crocuta*, while the image to the right shows vigilance behaviour of a black‐backed jackal towards a lion, *Panthera leo*, sleeping in the background.

Co‐detection rates for lions, jackals and brown hyaenas increased as the carcass age increased while spotted hyaena co‐detection numbers did not significantly change. Spotted hyaenas are able to utilise a carcass at all stages, they facilitate the opening of carcasses and also break down bones and skin when carcasses are completely consumed (White & Diedrich, 2012). During the intermediate phase of carcass decomposition, more of the soft tissue is accessible, allowing for multiple points of access for species to feed simultaneously. Although lions exhibited moderate levels of circadian activity overlap with sympatric carnivores, the co‐detection rates between lions and other species were virtually zero despite increasing with carcass age, suggesting lions almost entirely dominated resources when present. Our results are in accordance with those of Morris et al. (2023), who also found that lions dominated elephant carcasses in Tsavo East National Park, Kenya. Lions exhibit greater selectivity for larger carcasses and will often exclude competing carnivores from accessing these resources (Amorós et al., 2020). Similar to Amorós et al. (2020), co‐detection of spotted hyaena and lion was minimal in Madikwe and spotted hyaena appeared to use temporal partitioning to access carrion resources when lions were absent. In Madikwe, although co‐detection occurred, lions were the only species with which jackals did not feed on the carcass simultaneously. Jackals remained at the carcass, highly vigilant but did not feed when lions were feeding. If lions were resting nearby, jackals would feed but frequently paused to reassess the lion's location, indicating potential interference competition by lions with sympatric carnivores (Figure 4).

These findings support the theory that lions, like other large predators e.g. tigers, establish a landscape of fear in the proximity of carcasses (Curveira‐Santos et al., 2021; Panda et al., 2023; Ramesh et al., 2017), although jackals had high co‐detection rates, they were rarely detected with lions in comparison to other species. In all instances, elephant carcasses became available when other natural prey was less abundant (prior to the lambing season for prey species such as impala, *Aepceros melampus*, and springbok, *Antidorcus marsupialus*). Thus, during the periods when prey is less abundant, jackal may be more reliant on carrion for survival, resulting in higher risk tolerance and increased co‐detection rates with species, such as lions, that they would typically avoid.

Suppression of subordinate carnivores at carcasses has been observed in previous studies investigating carcass access and interactions (Allen et al., 2015; Bell et al., 2023; Panda et al., 2023). Our results show that these dynamics are further influenced by the diversity of species occurring in the area, as this is the first study to assess carcass use in an area with an intact carnivore guild. Sievert et al. (2023) recorded observations of civets at multiple carcasses of varying sizes in Liwonde National Park, Malawi, where predators occur in relatively low densities. Civets are not uncommon in Madikwe and could reasonably be expected to access carcasses as was the case in Liwonde National Park (Sievert et al., 2023). However, with only a single observation of a civet across all carcasses, it suggests dominance over mesocarnivores which occur at low densities when the carnivore guild is intact. Jackals are extremely flexible in their coexistence behaviours (Welch et al., 2022) and therefore the effects of dominance may not be as prominent when considering jackal co‐detection. This provides important insights into considerations of the effects of true subordinate carnivore suppression in the presence of intact carnivore guilds.

Despite studies suggesting suppression of brown hyaenas by spotted hyaenas (Hofer & Mills, 1998; Mills, 1984; Mills & Funston, 2003; Williams et al., 2021), we found high circadian activity overlap, virtually the same peak activity period (spotted hyaena; 21:00 h and brown hyaena; 22:00 h), and moderate levels of co‐detection between the two species. Although Mills (1984) suggested that low densities of brown hyaena were attributed to the more dominant spotted hyaena, in fenced reserves high densities of both species are common (Honiball, 2021; Vissia et al., 2023). The high population densities observed for spotted (~12.61/100km^2^) and brown hyaena (~14/100km^2^) in Madikwe (Honiball, 2021) can be understood by their seemingly tolerant behaviour of each other at shared resources. Therefore, despite spotted and brown hyaena having high dietary overlap (Honiball et al., 2021; Vissia et al., 2023), an understanding of feeding behaviours such as temporal use of shared resources may be more indicative of potential competition between carnivores.

This study provides unique insights into effects of an intact carnivore guild on how resources are shared and how subordinate carnivores adjust their behaviours at ephemeral resources according to the landscape of fear or through tolerance of a potential threat. We emphasise the importance of understanding intraguild dynamics at shared ephemeral resources and how this can alter our current perception of competition in the carnivore guild. As fenced protected areas become important sites for intact carnivore guilds globally, it is critical to understand all aspects of coexistence and, by investigating the role of scavenging, provide unique insights into these adaptations. We recommend further research into the behavioural adaptations of species consuming carrion at carcasses to enable coexistence in landscapes with intact carnivore guilds. Understanding these behavioural adaptions is critical for management as interactions between species at ephemeral resources may influence disease ecology, seasonal hunting pressure or survival of subordinate species as they increase the risk of exposure at these ephemeral resources. Furthermore, in areas where elephant densities are high, the implications of megaherbivore carcasses being a reliable food source need to be understood for food web ecology and community dynamics. Dominant predators efficiently utilised elephant carcasses in Madikwe and this could have effects on improved fitness due to the high energetic gains associated with scavenging (Brown et al., 1999). Reserves such as Madikwe could thus sustain higher densities of dominant predators should these ephemeral resources become consistent annual food sources, making these populations important for species survival and conservation efforts. This potentially reliable resource should therefore be factored into management models in areas with high elephant densities.

## AUTHOR CONTRIBUTIONS

**Terry‐Lee Honiball:** Conceptualization (equal); data curation (lead); investigation (lead); methodology (lead); visualization (lead); writing – original draft (lead); writing – review and editing (equal). **Robert S. Davis:** Conceptualization (equal); investigation (supporting); supervision (equal); writing – original draft (supporting); writing – review and editing (equal). **Liyabona Ntlokwana:** Conceptualization (supporting); data curation (supporting); investigation (supporting); writing – original draft (supporting). **Jan A. Venter:** Conceptualization (equal); project administration (lead); resources (lead); supervision (lead); writing – review and editing (supporting).

## CONFLICT OF INTEREST STATEMENT

The authors have no competing interests to declare.

## Data Availability

Data and code can be accessed through Mendeley Data via: Honiball et al. (2023), https://doi.org/10.17632/4s3fb7rcr5.1.

## APPENDIX 1

### 1.1

A description of the model used to determine if the length of time a carcass was monitored affected the probability of recording species co‐detection and the associated result. The model was applied to focal species in general and to individual species.

**Table:**

Model	Species	*β*_occasion_	Standard deviance	*p*‐Value
lm(Spp_codetection_probability ~ Monitoring_length)	All (lion + Spotted_hyaena + Brown_hyeana + Jackal)	.012	0.009	.195
	Lion	.017	0.011	.258
	Spotted hyaena	.04	0.027	.201
	Brown hyaena	.025	0.026	.383
	Jackal	.033	0.025	.248
